# Avascular Necrosis Bone Complication after Active COVID-19 Infection: Preliminary Results

**DOI:** 10.3390/medicina57121311

**Published:** 2021-11-30

**Authors:** Adam Sulewski, Dominik Sieroń, Karol Szyluk, Mikołaj Dąbrowski, Łukasz Kubaszewski, Dawid Lukoszek, Andreas Christe

**Affiliations:** 1 Adult Spine Orthopaedics Department, Poznan University of Medical Sciences, 61545 Poznan, Poland; mdabrowski@ump.edu.pl (M.D.); zaklad.sbk@ump.edu.pl (Ł.K.); 2 Division of Magnetic Resonance Imaging, Silesian Center for Heart Diseases, 41800 Zabrze, Poland; dominik.sieron.ch@gmail.com; 3 Department of Physiotherapy, Faculty of Health Sciences in Katowice, Medical University of Silesia in Katowice, 40752 Katowice, Poland; karol.szyluk@sum.edu.pl; 4 I Department of Orthopaedic and Trauma Surgery, District Hospital of Orthopaedics and Trauma Surgery, Bytomska 62 Str., 41940 Piekary Śląskie, Poland; 5 Dawid Lukoszek Physiotherapy Osteopathy, 42690 Hanusek, Poland; kontakt@dawidlukoszek.pl; 6 Department of Radiology, Inselspital, Bern University Hospital, University of Bern, Freiburgstrasse 10, 3010 Bern, Switzerland; andreas.christe@insel.ch

**Keywords:** avascular necrosis bone, osteonecrosis, SARS-CoV-2 infections, corticosteroids

## Abstract

*Background and objectives*: The course of SARS-CoV-2 (COVID-19) is still under analysis. The majority of complications arising from the infection are related to the respiratory system. The adverse effect of the viral infection on bone and joint tissue has also been observed. *Materials and Methods*: We present a group of 10 patients with degeneration of large joints and adjacent epiphyses of long bones and the spine, with a background of bone infarctions and avascular necrosis (AVN) immediately after infection with the COVID-19 virus. In MR imaging, changes in the characteristics of AVN were documented. *Results:* Observation of this group showed a clear correlation among the history of COVID-19 disease in the patients, moderately severe symptoms, high levels of IgG antibodies, and the time of occurrence of joint changes. No other clinically significant complications were observed following COVID-19 infection in the study group. No other risk factors for AVN or autoimmune or degenerative diseases were found in the study group. The group of patients responded well to empirical treatment with steroids, which normalized acute inflammatory symptoms and pain in the joints. *Conclusions:* During coronavirus (COVID-19) infection, there are complications in the locomotor system, such as microembolism and the formation of AVN; hence, more research is needed.

## 1. Introduction

The coronavirus 2 (SARS-CoV-2) (COVID-19) pandemic has stimulated an unprecedented response by the global scientific community to better understand the disease. However, many questions about SARS-CoV-2 remain unanswered. Various hypotheses have been formulated in regard to its pathogenetic mechanisms and treatment [1]. A plethora of reports on the long-term consequences of the infection, which also include the musculoskeletal system, have been published [2].

Systemic inflammation may play a role in the physiology of bone and joint tissue in COVID-19 patients [1]. Cytokines that are induced by COVID-19 include CXCL10, IL-17, and TNF-alpha [2]. They are responsible for reducing the proliferation and differentiation of osteoblasts.

Corticosteroids administered to most patients treated for COVID-19 in hospital also have an adverse effect on bone tissue [3,4].

In addition, single nucleotide polymorphisms in various genes encode for proinflammatory proteins, such as IL-1b, IL-6 and IL-8, which may affect biological activity and contribute to hypercoagulability in COVID-19 patients, thereby increasing the risk of bone necrosis [5]. The combination of hypercoagulability, leukocyte aggregation and vasculitis can impair blood flow in the blood vessels of the bone and contribute to the development of bone necrosis [5].

## 2. Materials and Methods

### 2.1. Patient Selection

Inclusion criteria: PCR indicating positive COVID-19 infection and joint pain during the course of the disease.

Exclusion criteria: prior injury to the affected joint, prior treatment with steroids, and severe chronic illness (DM and hypertension were not exclusion criteria).

### 2.2. Study Group Characteristics

The study included a group of ten patients who developed symptoms of joint dysfunction, which were classified as avascular bone necrosis in COVID-19 on MR images [6].

The criterion for classifying the severity of COVID-19 infection was defined according to a 4-point scale: mild, moderate, severe and critical (Table 1) [7].

The examined group of patients had not previously received any treatment for diseases of the musculoskeletal system (e.g., steroids), did not suffer from significant injuries or did not suffer from significant joint pain.

All the patients had a mean IgG and IgM COVID-19 antibody titer corresponding to the typical course of COVID-19 infection. Basic immunohistochemical tests were performed in all patients to rule out autoimmune diseases. HLA-B27 was negative in all patients. The examination of the synovial fluid in all patients revealed changes in the characteristics of aseptic arthritis.

### 2.3. Radiological Imaging Technique

The MRI consisted of (fat suppressed)-T2, pre- and postgadolinium T1-weighted imaging.

The MR images demonstrated bone lesions characteristic of AVN:

- T1 FSE: the initial specific findings are areas of low signal representing edema, which can be bordered by a hyperintense line, which represents blood products;
- T2 FR FSE: This may show a second hyperintense inner line between normal marrow and ischemic marrow. This appearance is highly specific for AVN of the hip and is known as the “double line sign”.

### 2.4. AVN Classification

Avascular bone necrosis was described using the Steinberg classification. The described changes also included subchondral infarctions with the involvement of articular cartilage (grade III) [8].

## 3. Results

The mean age of the patients was 61 years and six women and four men were included. The affected joints were three hips, four knee, a shoulder, a sacrum and a spine; four were treated for joint inflammation with steroid drugs and six were not.

The course of infection was mild in three patients, moderate in five and severe in two patients. Four patients were treated with steroid therapy (6 mg/day dexaven).

Clinical signs of musculoskeletal symptoms occurred 7 to 22 days from infection onset (mean 14 days) and appeared 5–10 days (mean 6 days) after the resolution of acute respiratory symptoms and elevated body temperature.

Patients were initially treated conservatively: non-steroidal anti-inflammatory drugs (NSAIDs), intra-articular steroid injections and therapeutic aspiration of the synovial fluid were implemented. There was no significant improvement.

Steroid therapy was initiated with an oral dose of dexamethasone 2 × 8 mg daily for a period of 2 weeks.

As a result, 3 out of 10 persons required arthroplasty and showed a good clinical outcome. One patient had chronic pain in the affected joint and is currently being treated conservatively; destruction of the joint surface was not shown in the control tests. In the remaining six people, there was no deviation in the control test follow up (Table 2).

### 3.1. Case Presentation

#### 3.1.1. Case 1 (Patient No. 5)

**Figure 1:**
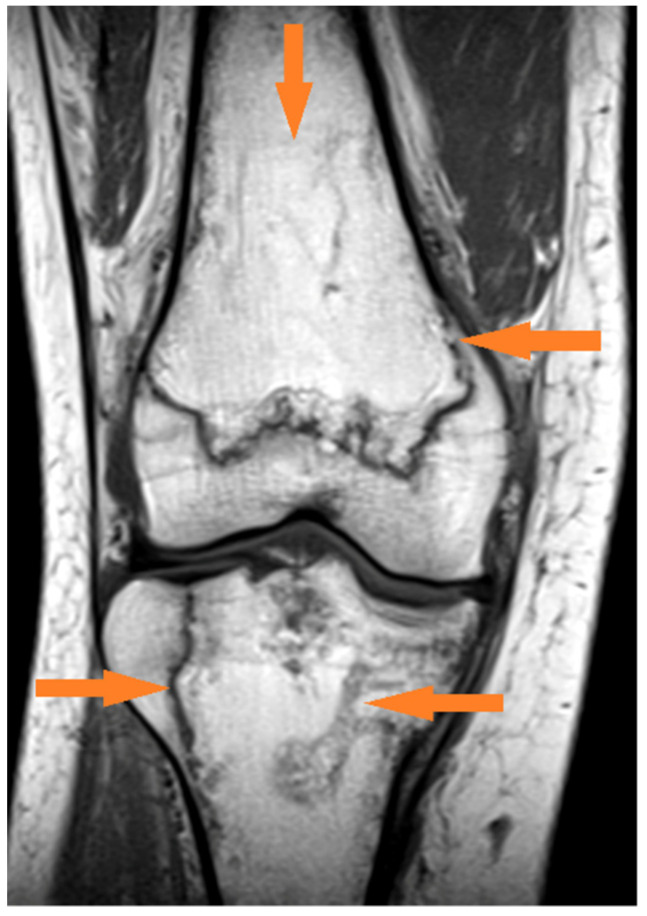
Changes found in the knee joint on T1-weighted sequence in a 43-year-old woman: geographical demarcation of bone infarction in the femur and tibia (orange arrows).

Patient No. 5 was a 43-year-old female nurse. The infection with SARS-CoV-2 was probably the result of contact with an infected patient at work. She was not ill at the time of infection, had no chronic diseases, was not taking drugs on a permanent basis, and had 1st degree obesity with a BMI of 32.0. The course of the infection was mild, fever symptoms were at the level of 38.5 Celsius, and she experienced coughing and weakness. Symptoms disappeared after approximately 5 days, she did not require hospitalization, and she recovered from the disease at home. After 21 days from the beginning of the infection, there was acute pain in both knees and joint swelling with joint exudate. She was treated orthopedically: aspiration of synovial fluid and non-steroidal anti-inflammatory drugs for 10 days showed no significant improvement. Magnetic resonance imaging of both knees was performed 21 days after the infection; numerous bone infarctions were revealed and features of aseptic osteonecrosis were grade 2 according to the Steinberg scale (Figure 1). The patient was admitted to the rheumatology ward, and steroid therapy was introduced (2 × 8 mg dexamethasone IV). After 2 days, the symptoms of pain and joint effusions began to subside. During the hospitalization, rheumatological diseases were excluded. Knee pain symptoms disappeared completely 10 days after the initiation of treatment with steroid drugs. The patient was monitored in an orthopedic clinic. Currently, 10 months have passed, and there are no abnormalities in the examination of the knee joints. A control magnetic resonance imaging was performed, which did not reveal any significant deviations from the norm.

#### 3.1.2. Case 2 (Patient No. 1)

**Figure 2:**
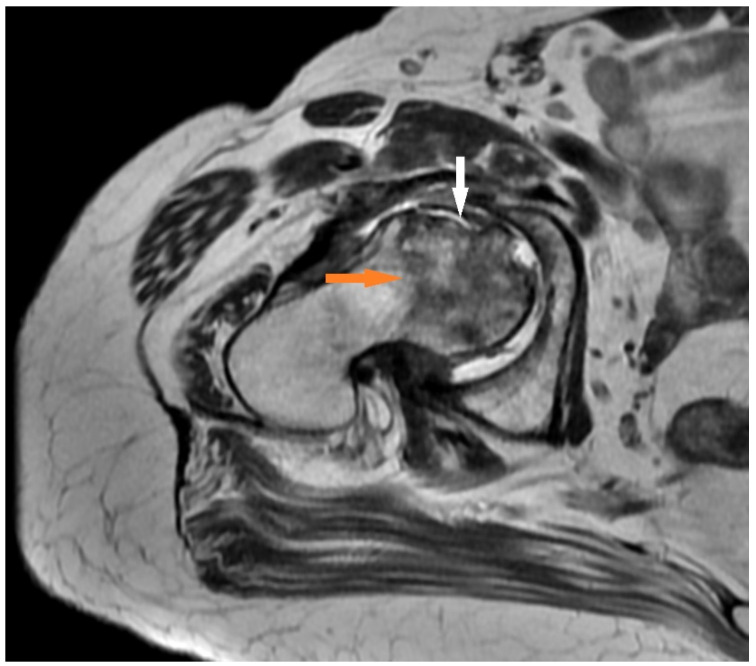
Changes found in a T2-weighted sequence in a 62-year-old man: delineation of the necrosis towards the femoral neck (orange arrow) and destruction of the anterior femoral head with impression of the cortical bone (white arrow).

Patient No. 1 was a 62-year-old male with chronic diseases (diabetes and hypertension). He was not suffering from joint pain and carried out light physical work. COVID-19 infection was moderately severe. He was hospitalized for 5 days and required oxygen therapy for 2 days. Symptoms of the infection resolved after approximately 11 days without steroid medication. Other disorders of the central nervous system included COVID-19 fog. Eleven days after the onset of the infection, an acute pain syndrome of the right hip appeared, as well as pain in the groin and thigh, which was intensified through weight bearing. The patient was treated with NSAIDs by the GP, and he saw an orthopedic specialist after 2 months. Magnetic resonance imaging was performed. Aseptic necrosis of the femoral head was visualized with the destruction of the articular surface and deformation of the femoral head (Figure 2). The patient was referred to the hospital for hip arthroplasty.

#### 3.1.3. Case 3 (Patient No. 7)

**Figure 3:**
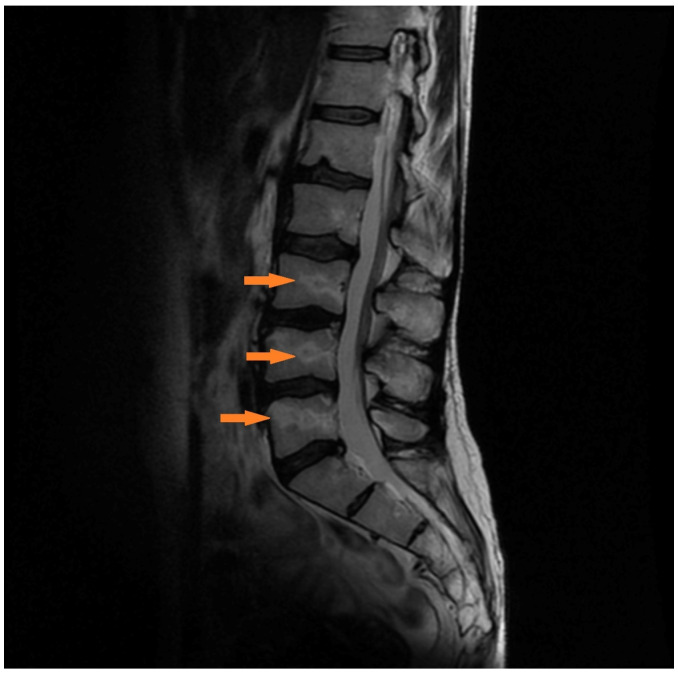
Hyperintense geographic edema in the lower lumbar on T2-weighted sagittal MR images, representing beginning bone necrosis. Delineation of the necrosis towards the vertebral body (orange arrow).

Patient No. 7 was a 66-year-old woman. The course of the infection was severe, and she had no chronic diseases. She required hospitalization for 2 weeks and oxygen therapy for 10 days without intubation. After the COVID-19 infection, there were complications in the respiratory system in the form of reduced respiratory efficiency, and she is currently undergoing further respiratory rehabilitation. Acute lower back complaints appeared 14 days after the onset of COVID-19 infection. For this reason, she consulted an orthopedic surgeon. There were no neurological deficits. Magnetic resonance imaging was performed, which showed numerous bone infarctions and features of aseptic necrosis of the lumbar spine (Figure 3). After treatment with steroid therapy with 2 × 8 mg dexamethasone IV, the symptoms of the spine disappeared after 2 days.

## 4. Discussion

Complications following COVID-19 infection are the focus of numerous clinical trials [9,10,11,12,13]. Pathological changes following COVID-19 infection have also been described in the locomotor system [1]. In our study, the formation of changes within the bone, with a background of AVN, was observed among the group of patients.

COVID-19 infection led to severe changes and dysfunction in the musculoskeletal system within 7–22 days after infection. The patients responded well to steroid therapy, while patients with advanced AVN (Steinberg grade 4) underwent arthroplasty. Four out of ten patients were treated with steroids in connection with COVID-19 treatment anyway.

### 4.1. Pathophysiology of MSK Diseases after COVID-19

A similar etiology of vascular and embolic changes over the course of COVID-19 infection has also been described in organs outside of the respiratory system, such as multiorgan failure, acute cardiac injury, cerebrovascular diseases, acute kidney injury, liver dysfunction, and venous thrombosis [14]. Undoubtedly, exacerbation of underlying diseases by SARS-CoV-2 infection also tends to worsen bone metabolism [15,16].

ACE2 deficiency, caused by viral invasion, can lead to bone matrix degradation [16]. Given that coronaviruses cause pneumonia and infection of the upper respiratory tract via ACE2 receptors in ATII cells, ACE2-dependent effects on bone tissue should also be noted. ACE2 is a potential factor that regulates bone biology during COVID-19 infection [17,18].

Bone complications from infections or treatments are likely to emerge in the next few months, similar to the SARS outbreak in 2002–2003. At that time, reports of joint pain, decreased bone mineral density (BMD), and necrosis of femurs and tibias could only be partially explained by high-dose steroid treatment [19]. Another in vitro study showed that the specific SARS-CoV protein, 3a/X1, directly promotes osteoclastogenesis, thereby accelerating osteoclast differentiation from monocyte/macrophage precursors and increasing the expression of the NF-kB ligand receptor activator (RANKL) and inflammatory cytokines, such as TNF-α, which indirectly promote osteoclastogenesis [20,21].

### 4.2. Corticosteroids Therapy

This study observed people with symptoms of AVN after a history of COVID-19 without steroid therapy. The first symptoms appeared on average 14 days (range 7–22 days) after infection. Probably in our patient group, steroid therapy did not directly influence the development of AVN. One study reported that symptoms of AVN appeared 58 days (range 45–67 days) after infection with COVID-19 [20]. However, the risk of AVN after steroid therapy ranges from 6 months to 1 year [20]. There is a lack of consensus about the dose and duration of corticosteroid treatment as a risk factor for developing AVN. One prospective study found that the risk of AVN increases significantly with the dose of >20 mg/day [22]. Our patients used 6 mg/day. COVID-19 disease appears to be an independent risk factor for AVN and possibly accelerates the risk of AVN after a history of COVID-19 treated with steroid therapy.

In the case of COVID-19, corticosteroids were primarily considered as a way to contain this “cytokine storm” and its aftermath: ARDS, disseminated intravascular coagulation, and shock [20]. This usually occurs within the first 8–15 days of infection [23]. Treatment with steroids is attempted, especially at the onset of dyspnea, or even earlier, to prevent the progression of the “cytokine storm” [21]. The anti-inflammatory properties of corticosteroids reduce systemic inflammation and exudates in the lung tissues, and prevent further diffuse alveolar damage, thereby improving hypoxia and minimizing the risk of respiratory failure [16]. Most of the studies on the use of corticosteroids to treat COVID-19 have shown variable results, but this is mainly due to a marked heterogeneity in the research methodology [15].

### 4.3. Clinical Reports

In the examined group of patients, no risk of bone changes in relation to the general condition of the patient and the severity of the course of COVID-19 disease was observed. For three patients of our observed group, the occurrence of AVN, with the consequent destruction of the articular surface and permanent changes (joint damage), was observed. These patients were treated with arthroplasty in relation to their hip joints. In the remaining seven patients, complete remission of the changes was observed after the steroid drugs, without permanent sequelae. However, the long-term consequences of bone changes over the course of COVID-19 are not known, as our observation period did not exceed several months.

AVN is a known complication after steroid treatment of severe COVID-19 infections or in long COVID-19 infections [20,24,25]. We described 10 cases who suffered from AVN shortly after a COVID-19 infection without prior steroid treatment. Apparently, COVID-19 infection alone may represent a risk factor for developing AVN. On average, AVN begins 2 weeks after COVID-19 onset in contrast to long COVID-19 late-onset AVN [20].

The following differential diagnoses should be considered in an individual with signs or symptoms suggestive of COVID-19-related AVN: bursitis or tendinitis, chondral damage or loose bodies, stress fracture, labral tear, muscle strain, neoplasm, psoriatic arthritis, rheumatoid arthritis, septic arthritis, Paget’s disease, piriformis syndrome, sacroiliac dysfunction, and radiculopathy [26].

### 4.4. Limitations

However, we are aware of limitations that may interfere with the results. First of all, the small size of the group and the resulting inability to divide into groups determined e.g., the affected area (knee, hip). At the present stage, the small size of the group makes it impossible to standardize the recommended management of AVN in the course of COVID-19. Additionally, the coincidence of AVN occurrence in this group may also be present. The small size of the group made it impossible to apply precise inclusion and exclusion criteria. However, we believe that the preliminary report will allow more attention to be paid to osteoarticular problems that may be associated with AVN treatment as a complication of COVID-19.

## 5. Conclusions

SAR-CoV-2 can affect bones presenting with symptoms 1–3 weeks after infection. This may resolve with medical management or result in end stage AVN that responds well to arthroplasty.

## Figures and Tables

**Table 1:** Characteristics of patients.

Patient No.	Age	Sex	Chronic Diseases	BMI	Severity of COVID-19	COVID-19 Therapy/Steroids
1	62	M	DM	23	severe	no
2	56	M	DM	30	mild	no
3	57	F	no	26	severe	yes
4	70	F	no	25	moderate	no
5	43	F	Hypertension	26	moderate	yes
6	54	M	Depression	22	moderate	yes
7	66	F	no	20	moderate	no
8	39	F	no	19	severe	yes
9	68	F	no	26	mild	no
10	73	M	no	27	moderate	no
mean SD	58.8 11.3			24.4 3.37		

**Table 2:** Characteristics of joint lesions in patients with COVID-19.

No.	Joint	Time of Onset of Joint Symptoms from the Beginning of Infection	Arthroplasty	Steinberg Scale	Follow-Up (Months)	VAS Pain Initially	Pain Follow Up VAS
1	hip	11	yes	4	10	8	2
2	knee	10	no	2	9	9	0
3	shoulder	11	no	2	7	6	0
4	hip	7	yes	4	7	8	0
5	both knees	21	no	2	8	7	1
6	knee	17	no	3	10	8	0
7	lumbar spine	14	no	2	8	8	1
8	sacrum	14	no	2	5	8	1
9	hip	22	no	2	5	7	0
10	knee	17	yes	4	4	8	0
mean		14		3	7	8	0,5

## Data Availability

Data are available upon special request.
